# Tics, Tourette’s and Related Muscle Pain in Children: A Review

**DOI:** 10.3390/muscles5010012

**Published:** 2026-02-09

**Authors:** Stuart Evans

**Affiliations:** School of Education, La Trobe University, Melbourne 3086, Australia; stuart.evans@latrobe.edu.au

**Keywords:** Tourette’s, motor tics, phonic tics, muscle injury, Tourette Syndrome

## Abstract

Tourette Syndrome (TS) is a neurodevelopmental disorder depicted by the occurrence of tics and accompanying behavioral problems that commonly appear during childhood. Tics, both motor and vocal, may cause musculoskeletal pain. Both acute and chronic muscle pain have been recognized as a common comorbid aspect of TS-related tic disorders in childhood. The pain most reported in children includes cervical, throat, shoulder, ocular, and joint pain, with most children reporting musculoskeletal pain in more than one part of the body. The impact of muscular pain caused by motor and phonic tics can negatively affect a child’s quality of life. This review describes the association and causation of musculoskeletal pain in childhood tics and TS, which are commonly under recognized and diagnosed. An analysis of the presence of musculoskeletal pain, the severity of the pain, the location of the pain and the movement incapacity due to pain in children is reviewed. Pharmacological and non-pharmacological interventions known to improve musculoskeletal pain in children are highlighted with supportive frameworks evaluated. Further research is needed to better understand musculoskeletal pain cause(s) and prevalence along with age-appropriate assessment methods and outcomes measures. Motor- and phonic-related musculoskeletal pain should be recognized as a common comorbid characteristics of TS and tic disorders in childhood. Such recognition may lead to greater therapeutic opportunities for this problematic condition.

## 1. Introduction

Tourette Syndrome (TS) is classified as a neurodevelopmental disorder that is characterized by co-occurring motor tics that often occur with a singular vocal tic. These tics are said to happen frequently throughout the day or periodically for 12 months after the first episode of the disorder. The progression of TS and the manifestation of motor and/or phonic tics are classified as movement disorders. Movement disorders are generally classified as a disorder of the central nervous system (CNS) in that they result in unusual and/or unwanted movements. These movements are commonly distinct from muscular weakness or muscular spasticity [1]. Dysfunction of the basal ganglia and frontal cortex play an important role in most movement disorders in children [1].

Movement disorders are generally separated into two classifications. The first classification is hyperkinetic movement disorders, related to excessive movement (e.g., extreme, unnatural, involuntary and relatively spontaneous movement). Movements encompass tics, stereotypies, chorea, myoclonus, dystonia, and tremors [2]. The second classification comprises hypokinetic movement disorder that relate to a paucity of movement (e.g., diminished amplitude, reduced speed, or loss of movement), including bradykinesia, akinesia, and rigidity [2]. In contrast to hypokinetic movement disorders, hyperkinetic movement disorders, notably tic disorders, are comparatively frequent in the pediatric population [3]. The most prevalent pediatric movement disorders are tic disorders, commonly associated with TS [2].

In 1825, Jean-Marc Gaspard reported the case of a French noblewoman who presented with involuntary body movements of the shoulders and neck combined with vocalizations, including dog barking sounds and the repeated use of obscene language. Subsequently, George Gilles de la Tourette reported nine patients with tic disorders [2]. A tic was, initially at least, considered to be an indication of a functional movement disorder such as neurosis, vanity and egotism. However, advances in medical knowledge have resulted in tics being generally mentioned in a neurobiological context. These are regularly accompanied by psychiatric comorbidities such as attention-deficit hyperactivity disorder (ADHD), obsessive–compulsive disorder (OCD), anxiety, and depression [3].

Common evidence points to both TS and tics commencing in childhood, although both can diminish within one year. Nevertheless, some motor tics can persevere and cause various problems for children, including physical discomfort, which may interfere with daily activities and school performance. Unfortunately, misunderstanding and misconceptions of TS and tic disorders remain [3].

As the severity, frequency and duration of tics vary, the level of musculoskeletal pain will differ in each child. Pain is more prevailing among TS sufferers compared to the general population and can negatively impact a child’s quality of life [4]. Identification and clinical diagnosis are summarized as the presence of motor and phonic tics that appear during childhood (prior to 18 years of age) with a progression exceeding one year. The American Psychiatric Association’s Diagnostic and Statistical Manual of Mental Disorders, 5th edition (DSM-5) details five tic conditions:

1. Provisional tic disorder.
2. Continual (chronic) motor or vocal tic disorder.
3. Tourette’s disorder (TD).
4. Other specified tics disorders.
5. Unspecified tic disorders [5].

Yet, despite if, and when, a clinical diagnosis is made, the concerns, implications and complications related to musculoskeletal pain in children are seldom considered. Therefore, this review examines the literature as to the mechanisms that cause motor and/or phonic tics in children diagnosed with TS and their effect on musculoskeletal pain and musculoskeletal injury. Moreover, the present review focuses on current treatment options that are accessible for children as well as looking into what future treatments could entail. After highlighting the limitations in many of the prevailing studies available so far, a holistic and multidisciplinary approach that integrates modern technological advances that could help monitor and temper children’s tics, and the related musculoskeletal pain is explored.

### 1.1. Scope and Structure of This Review

In this review, common neurological, neurodevelopmental and neuromuscular mechanisms related to TS and the consequential motor tics and/or phonic tics and their impact on musculoskeletal pain and potential muscle injury (muscle damage) in children are categorized. For this review, children are defined as those in the primary (elementary) and middle school years, ranging from 5 to 12 years. The impact of TS and both motor and phonic tics are emphasized along with potential causes of musculoskeletal injury and muscle function. A focus on TS, motor and phonic tics and their impact on functional movement, incorporating gross and fine motor skills in children, is also reviewed. Current developments in medical and therapeutic interventions, including non-pharmacological and lifestyle-based approaches and pharmacological interventions that target the restoration of musculoskeletal function in children diagnosed with TS and motor and/or phonic tics are included.

### 1.2. Etiology

The etiology of TS and the associated motor and phonic tics are multifaceted and likely encompasses an amalgamation of genetic, neurobiological, behavioral and environmental factors. Differences in understanding the cause of TS and tics in children endure, including the factors contributing to the variability in clinical indicators in how to treat TS, tics and the comorbidities, including musculoskeletal pain. Nevertheless, a tic is classed as simple or complicated (complex) and involves groups of muscles in the head, neck, trunk and/or extremities (upper and/or lower) [6]. Simple motor tics, such as clonic tics, are said to include one group of muscles only [7]. Dystonic tics are terse abnormal postures that are relatively constant. Tonic tics are isometric contractions of a group of muscles [7]. The beginning of motor tics can be relatively simple such as when a child blinks or sniffs repeatedly. In contrast, complex motor tics include relatively synchronized movements, including that of the neck. In some scenarios, muscular motor tics can involve unusual facial expressions, shoulder shrugging, head jerking, grimacing, head tossing or other movements. A clonic, rhythmical tic may resemble a tremble or myoclonus [7]. Incidences of simple dystonic tics encompass blepharospasm (forced eye closure), oculogyric movements, bruxism (teeth grinding), sustained mouth opening, paroxysmal torticollis, dystonic neck movement, shoulder rotation, or tensing of abdominal or limb muscles [7]. A persistent blocking tic (dystonic and/or tonic) and the resultant muscle contraction may inhibit activities of daily living due to the subsequent impact on the motor control system. In many situations, children may duplicate a specific movement or action to alleviate the impulse until “it feels good.” This feeling can relate to compulsive behavior and may be interpreted as an incontrollable tic [8]. However, in some situations, it is possible for a child to suppress a tic and retain a modicum of control over muscle contractions. Nonetheless, there is downside, as persistent tic inhibition may be followed by a tic rebound. A rebound is when a child experiences a transient increase in tic magnitude and frequency [9]. While the frequency and duration of tic suppression and tic rebound in children are recognized, the magnitude and consequential bearing on both motor control, movement control and musculoskeletal pain are largely unresearched areas. What is commonly stated in the literature, however, is that due to the discernible disposition of tics, some children diagnosed with TS who have a tic disorder are often on the receiving end of unnecessary and sometimes harmful attention from others. This can lead to bullying, a loss of confidence and self-esteem and a lack of motivation to pursue conventional child-centric pursuits. Moreover, a child can further attempt to suppress their tics in social situations to counteract and cope with possible negative attention [10].

Researchers have approximated that an international prevalence of 0.6–1% exists in schoolchildren diagnosed with TS and a tic disorder [11]. The researchers note that this is 3–4 times more frequent in boys than in girls. Data from the 2007 National Survey of Children’s Health (NSCH) showed a projected occurrence of 0.3% among U.S. children aged 6–17 years [12]. Nonetheless, the proportion of this figure that relates to musculoskeletal pain, musculoskeletal injury or musculoskeletal damage in children is primarily unknown.

### 1.3. Diagnosis of Tourette’s Syndrome: Differential Process

Identification and diagnosis of TS and a tic disorder are frequently grounded on a child’s medical history. The American Psychiatric Diagnostic and Statistical Manual of Mental Disorders (DSM-5) [5] states that to establish TS, the tic (or tics) must have commenced prior to the age of 18 years with the child having had a tic (or tics) for at least one year. Moreover, the child will have comprised at least two motor tics and one phonic tic without regard for tic frequency [13]. Tics typically begin around 6–8 years of age, and 90–95% of TS cases have an onset of tics between the ages of 4–13 [8]. In instances in which severity persists into adulthood, tic symptoms are more severe than in childhood and are characterized by self-harmful tics or coprolalic utterances [8]. Both instances would have a consequential effect on the musculoskeletal system with possible muscle restriction, pain, or injury as the most extreme effect. Tics also demonstrate characteristics that distinguish them from other common childhood movement disorders such as stereotypies, choreas and dystonias. The distinctive features of tics comprise:

1. They intensify-and-decline in magnitude, severity and frequency.
2. The individual attribute of the child’s movement changes over time.
3. They are fleetingly suppressible.
4. They are typically associated with sensory phenomena (Table 1).

### 1.4. Classification of Tics

Tics are commonly actioned by their location (e.g., anatomical location) along with the frequency, intensity (forcefulness, muscular strength), and time and type (i.e., complexity). A frequently used rating scale is the Yale Global Tic Severity Scale (YGTSS), which contains an ordered methodology to help medical professionals classify tic severity [14]. The YGTSS includes distinct scores from 0 to 5 for number, frequency, intensity, complexity, and interference (the degree to which planned actions or speech are interrupted by tics) of both motor and phonic tics. However, since TS symptoms can and do vary in children, the variable nature of TS makes a diagnosis difficult for medical professionals.

**Figure 1:**
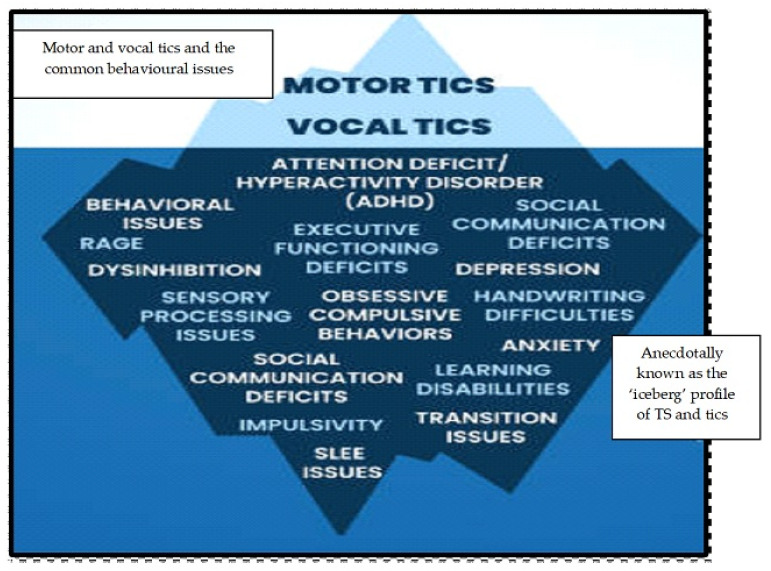
Motor and vocal tic and the tic pyramid.

The Tourette Syndrome Diagnostic Confidence Index (DCI) was created by an expert group of clinicians [13]. The DCI assigns a score from 0 to 100, which reflects the likelihood of a child having or ever having had TS [15]. Other rating scales comprise the Shapiro Tourette Syndrome Severity Scale, Tourette’s Syndrome-Clinical Global Impression Scale, and the Hopkins Motor and Vocal Tic Scale [16]. A characterization of a simple tic would involve the muscles of the eye lids, including the levator palpebrae superioris and the orbicularis oculi [13]. These tics are termed simple since they involve a single muscle contraction. For instance, simple tics can comprise muscles of the face responsible for lifting and everting the muscles of the higher lip (e.g., levator labii superioris, levator labii superioris alaeque nasi, risorius, levator anguli oris, zygomaticus major and zygomaticus [13]). Muscles responsible for depressing and everting the lower lip such as the depressor labii inferioris, depressor anguli oris and mentalis muscles are also impacted [17]. Taken together, the classification of TS, motor and vocal can also be associated with conditions that may accompany tic disorders include rage, anxiety, social communication deficits, obsessive-compulsive disorder (OCD) and learning disabilities. These comorbidities are sometimes known as the tic pyramid (Figure 1).

In many situations, a motor tic will characteristically progress in a rostral–caudal pattern, slowly becoming more complex. In this situation, muscle contractions behave in a stereotypical and repetitive way [8]. In contrast, a phonic tic tends to occur after the onset of a motor tic and progresses from conventional and somewhat predictable to complex vocalizations. Nevertheless, Jankovic [7] states that while a vague difference exists between tic classifications, the noises and expressions created are due to muscular contractions of laryngeal, respiratory, oral, or nasal musculature. Tic severity typically peaks at between ages 10–12 despite a reported decrease in symptoms during adolescence [18].

### 1.5. Motor Movement Execution and Measurement Considerations

Motor skills involve numerous muscle contractions depending on what skill is performed. Typically, motor skills are classified as either gross or fine. Gross motor skills denote large movements of the body that involve the use of major muscle groups whereas fine motor skills involve smaller and precise movements involving the fingers and toes [19]. The basal ganglia and the cortico–striato–thalamo-cortical (CSTC) circuitry are neurologically associated with fine motor skills [20]. Adequate fine motor skills are fundamental for children to learn from an early age. Fine motor skills help children manipulate smaller objects with their hands and fingers, for example, gripping, clutching, holding and pinching. Holding a pencil is one such example. Children continually acquire, refine and consolidate their motor functions and integrate these skills. Consequently, a deficit in fine or gross motor skills may negatively influence children’s motivation and self-esteem [19]. Taken together, dysfunctions concerning motor cortical areas, such as increased initiation of the premotor cortex and additional areas involved in the preparation and coordination of temporal movement and activity, are related to TS [21].

Some studies involving functional magnetic resonance imaging (fMRI) in adults with TS and tics have examined the process of movement [22,23], although results are inconclusive in children. fRMI is an imaging method that indirectly tracks neural activity via blood oxygenation levels. Or put simply, fMRI tracks blood flow to “see” brain activity. A study by [23] revealed that when children and adults refrain from making eye blinks, the frontal cortex and striatum were more activated in those diagnosed with TS compared to those without. The explanation given was that activation, or stimulation, of this control system may help preserve control over tics. The authors in [23] suggested that these control systems are continually active in those suffering from TS. Additional fMRI studies have attempted to understand more about TS and the muscular implications of tics by studying cognitive tasks, the hypothesis being that TS sufferers will have eccentric task control that is equivalent to a lack of control over unwanted motor and muscular movements. fMRI has examined the dynamic causal modeling of motor control in drug-naïve children with chronic tics [24]. The results showed heightened activation in motor and somatosensory regions and increased interhemispheric connectivity. However, notwithstanding deficits in motor inhibition, children performed well on motor tasks, suggesting compensatory neural adaptations.

Recent neuroimaging studies in children with TS have suggested that fine motor skills are impaired and are somewhat heritable [25]. This effectively means that a tic is an inherited trait. Nonetheless, the suitability of using neuroimaging and fMRI in children given their changing levels of maturation and developing musculoskeletal system makes studying them problematic [25]. For instance, intensified movement in children can increase during the scanning process due to apprehension, unease and anxiety that result in uncontrolled muscular movements that consequently reduce compliance during the imaging process [26]. An alternative technique is diffusion tensor imaging (DTI). DTI can determine the passage of water molecules through the brain. In the relatively small but increasing DTI literature, results indicate an increased role of the corpus callosum, somatosensory and motor cortex, and caudate [25]. Although a growing interest in DTI exists, many existing results relate to adults and not children. Comprehensive Behavioral Intervention for Tics (CBIT) [27] is another novel and emerging assessment and treatment option. This non-invasive methodology is centered on habit or trait reversal therapy. CBIT has demonstrated to be effective in some studies, but as acknowledged by the authors, the therapy is underutilized due to the absence of qualified clinicians that can provide the therapy.

### 1.6. Muscle Pain

Tics frequently lead to significant musculoskeletal pain, driven by the repetitive strain placed on joints and muscles [28,29]. Muscle pain is more ubiquitous among TS patients compared to those without it, negatively impacting quality of life [9]. However, clinical assessment of muscle pain can be subjective depending on the child’s pain tolerance level, making diagnosis problematic.

It is estimated that the prevalence of adults suffering from TS and associated muscle pain is approximately 47.5% [30]. Moreover, approximately 15–20% of children have reported discomfort as a conventional characteristic of tic disorders [30]. While many children reported musculoskeletal pain, neuropathic pain has also been reported. Moreover, pain was generally associated with voluntary movements or when a child attempted to suppress their tics [30], which can then result in soft tissue damage due to the forceful and uncontrolled nature of some tics [31]. Muscle tenderness is one reason why some children start pharmacologic interventions [32]. Notwithstanding a child’s individual musculoskeletal pain tolerance, most clinical ranking scales, questionnaires and outcome measures used in therapeutic interventions fail to contain information regarding the scaling of pain relative to children. To help address this issue, Green et al. [4] proposed that to standardize future research efforts, tic-related musculoskeletal pain should be classified according to (a) accompanying instantaneous pain, (b) accompanying delayed injury/pain, (c) suppression-related pain, (d) premonitory urge-related pain, and (e) related primary pain syndromes. Greater evidence-based knowledge would enable medical professionals to enhance multidisciplinary strategies to help children manage their condition. Moreover, in some scenarios, ongoing musculoskeletal discomfort may be a masked or hidden trait [33], yet, for others, it could be self-harmful (e.g., self-inflicted) or include headaches due to vigorous gyrations of the whole body [28]. Additionally, children have reported increased pain due to greater tic severity [34,35].

The interplay between tic severity, frequency, duration and the anatomical location and nature of muscle contractions are achieved through complicated neurological mechanisms and connections (Table 2). Recent studies [36] revealed that 87% of participants suggested that discomfort caused by tics impacted their everyday life, while [37] found that young children coping with tic-related pain sought parental support. In contrast, a teenager’s favored coping strategy was to isolate. Further research could advance knowledge of clinicians and healthcare professionals to improve the support available for children with TS and tics, including in-school support.

### 1.7. Location of Muscle Pain and Pain Descriptors

Of what scant literature exists concerning the location of muscular pain experienced by children due to TS and consequential tics, and aside from headaches and migraine related pain, the most reported musculoskeletal pain appears to be cervical, throat, shoulder, ocular, and joint pain. Conversely, research has ranged from investigating tic-related pain and pharmacological and non-pharmacological possibilities to help manage musculoskeletal pain in those aged 16–71 years [38] to novel three-day, community-based, holistic interventions for enduring musculoskeletal pain [33]. In describing musculoskeletal pain, the authors [33] observed that musculoskeletal pain can result in feelings of desperation, anguish and low mood states in children. Other reported symptoms included general muscle and throat discomfort, with damage to dentures amongst the frequently described injuries. Overall, the evidence points to musculoskeletal and joint pain as the most recognized in children [39,40].

The delicate balance of knowing if, and when, medication is required and is intricately connected to a child’s symptoms. While the principal aim of medication is to treat and manage the condition, ultimately, existing medication fails to get to the root cause. Research has also shown that when medication is used, it is frequently deemed ineffective [30]. Moreover, the type of medication prescribed differs and, therefore, is often accompanied by side effects [41]. This could influence adherence and decisions made about medication. Importantly, the medication that adults are free to consume is not necessarily prescribed to or endorsed for children, given that parental consent must be achieved and the dose/response must be age-appropriate.

**Figure 2:**
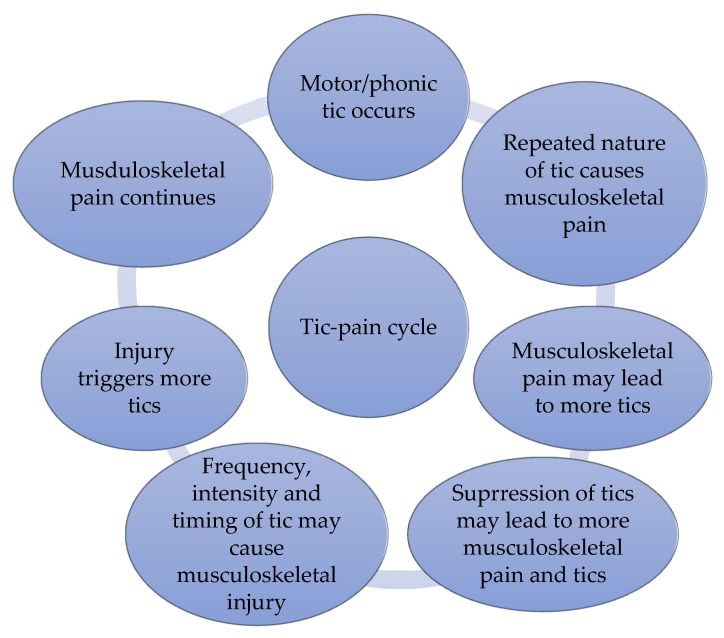
The “Tic-Pain Cycle”. Adapted from [38].

The repetitive nature of tics has led to a “tic-pain” cycle construct [35]. One example is a tic attack (abrupt spells of tics that last for several minutes or hours). Described as a cycle that is fashioned by varying physical and sensory sensations, cognitive elements, and anxiety-related beliefs, researchers [38] classified them into four themes: “The tic-pain cycle,” “The impacts of pain,” “The importance of support,” and “successfulness of pain management techniques.” Such multifaceted and meshed themes are based on a cause/effect relationship (Figure 2). While the authors concluded that the attributes of tics can be associated with musculoskeletal pain, inclusive of the applied effort needed to produce them, no firm conclusions can be made as the outcomes were based on an online survey that included cohorts aged ≥16 years.

### 1.8. Coping with Pain and Pain Management

Pain is a complex biopsychosocial phenomenon [42] and can have lasting effects. As well as managing tics, children can be self-dependent in finding ways to manage their condition. Additional options available for children include clinical assessment and different methods of treatment and support. However, this commonly means a long waiting time for children and parents to see a clinician that is often confounded with inadequate financial support. Subsequently, accessing professional and expert support and clinical care is often challenging [43]. Both pharmacological and non-pharmacological options are available as treatment options for children. While pharmacologic interventions remain a backbone of TS and musculoskeletal pain management, they are generally reserved for severe cases given their long-term side effect profiles [28]. Notably, no consensus exists in what medication should be given to children. When medication is prescribed, antipsychotic medication is the most effective and the most reportedly used for muscle pain management [44] with alpha-2 agonists are commonly selected for their safety [45]. Novel treatment modalities, including transcranial magnetic stimulation [46] and vagus nerve stimulation [45] are under investigation and remain experimental while osteopathic treatments have been effective at treating muscle pain [47]. However, despite its clinical relevance, osteopathy has not been adequately explored in children despite the treatment being a possible palliative non-pharmacological gap. Nevertheless, treatment of a child’s tic disorder commonly requires a multidisciplinary, allied-health approach involving general practitioners, dietitians, neurologists, psychiatrists, psychologists, behavioral therapists and school support aids. Arguably, there exists additional factors that warrant consideration in children compared to adults when treatment options are considered. For instance, sensitivity of musculoskeletal pain and discomfort may be influenced by the child’s age, gender, musculoskeletal and neuromuscular development and maturation stage and individual coping mechanism. Socio-cultural considerations also need consideration [48]. Likewise, the approaches used by children and adolescents to manage tic-related musculoskeletal pain appear to be rather innate and not necessarily based on the diagnosis of professional support. To illustrate, researchers have noted that pain coping strategies change with age [49] and that cognitive self-instruction and distraction-based strategies are habitually presented by younger children (6–12 years) [50]. The latter approach can also necessitate parental involvement if the child is struggling with pain.

## 2. Discussion

Tourette’s syndrome is a persistent condition which affects children’s everyday life. TS and the commonly associated tics occur through early childhood with varying levels of severity. A decrease in TS, tics and the accompanying musculoskeletal pain is common in adulthood, with approximately 40% eventually becoming symptom-free [50]. This indicates that children may develop variable coping and adjustable strategies combined with pharmacological and/or non-pharmacological treatment. Children’s experiences are frequently correlated with sensory experiences or phenomena such as vigorous urges and somatic hypersensitivity that are often as distressing as the tics themselves [13]. Yet, the significance of musculoskeletal pain and associative muscle damage in children and, importantly, the treatment and management of such musculoskeletal-associated injury remain under investigated. Due to the small number of prior studies that have explicitly examined muscle skeletal pain, muscle discomfort and/or muscle injury in children, it is difficult to obtain uniform information. This is specifically apparent when outcomes related to children’s conditions, their heterogeneity, their different manifestations and persistence of tics and the consequential muscle pain, their different diseases (when present) in comorbidity, and the exact category of pharmacological and/or therapeutic treatment (if prescribed) are scrutinized. Therefore, a combined neurobiological and neuromuscular framework that can rationalize the complicated neural dynamics underlying both the cause and the inhibition of TS, tics and muscle pain in children remain absent. While research (e.g., [51,52,53]) has observed lower intensities of muscular pain when tracked longitudinally, the neurological and neuromuscular causes remain unclear.

Musculoskeletal pain can influence a child’s mood and motivation, circadian rhythm and overall enjoyment. Recently, Małek [54] evaluated the severity of persistent, chronic pain in adults suffering from TS. The assessments included a Pain Coping Strategies Questionnaire, a short-form McGill Pain Questionnaire-2 (SF-MPQ-2), a Yale Global Tic Severity Scale (YGTSS) and Visual Analog Scale (VAS). Interestingly, 73.7% of respondents recounted that they had experienced tic-related pain, with many reporting pain in numerous anatomical locations. The current research primarily focuses on adults, with male and female differences often cited, yet the complex TS, tic and muscular pain system encompasses a broader range of issues in children, including the school environment, parental influence, psychosocial and psychological anxieties. A continuing concern is if information gained from self-reported questionnaires and scales can be used, if at all, to help children cope with muscular pain and discomfort. While some longitudinal studies that tracked the severity, the frequency, the timing and the specific type of musculoskeletal pain caused by tics exist, it remains uncertain as to how to best analyze, treat and help children. Consequently, a better and more targeted focus is needed, specifically throughout the pre-pubescent to pubescent years as motor and phonic muscular tics may wax and wane along with physiological and biological changes. Critically, inferred techniques to measure or infer pain indicate a child’s feelings and do not always imply or provide the extent of musculoskeletal damage [54]. The introduction of self-reflection and self-reporting in older children may help with understanding when, where and how pain arises. This may assist with non-pharmacological methods of tic-related coping approaches. Further consideration can also be placed on understanding abnormalities in the brain, particularly those associated with motor control. Attention should be focused on the basal ganglia–cerebellar–thalamo-cortical system [55], given the significance of the interpretation and modulation of pain experience [56,57]. Potential research, for instance, research using computational modeling, machine learning and generative AI, can be considered as future therapeutic interventions. Altered sensory gating and interoceptive processing abnormalities may also advance understanding concerning the nuances involved in the TS-tic relationship in children [55]. Herman [58] notes that distraction and cognitive self-instruction are more cognitively demanding but optimal for pain reduction for adolescents, while others have suggested that relaxation therapy is a recommended component [59]. Despite a relative plethora of treatments and interventions available, and notwithstanding the collective knowledge of TS, motor and phonic tics and the musculoskeletal pain experienced by children, there remains much to discover. Future technological innovation and interventions, both pharmacological and non-pharmacological, offer hope that motor and phonic tics in TS and the musculoskeletal pain experienced by children can be better explained, if not necessarily cured.

## 3. Conclusions

Musculoskeletal pain caused by motor and/or phonic tics in children should be recognized as a common factor in TS. There is an apparent relationship between musculoskeletal pain with the intensity and the severity of tics; that pain may hinder children’s ability to function and participate in daily activities. There has been significant research into both understanding the root cause or causes of TS and the associated tics and subsequent muscle pain as well as pharmacological and non-pharmacological options to effectively treat the condition. This is to ensure that the interdisciplinary medical teams are armed with the latest clinical and evidence-based information to help children and their families manage the condition, with a view to limiting the impact the disorder has on children’s activities of daily living. Assessment regarding the efficiency and effectiveness of pain relief procedures is another area to be explored in children. Holistic therapeutic interventions should expand their scope to consider options to mitigate muscle pain caused by tics, with future considerations given to the role of machine learning and generative AI in shaping long-term strategies that help children cope and perhaps reduce or eliminate tic-induced muscle pain.

## Figures and Tables

**Table 1:** Overview of tic disorders, criteria of tics and characteristics classifications of tics [5].

Tic Disorders	Diagnostic Criteria		Exclusion Criteria
Type	Tics Present	Duration of Tics	
Tourette syndrome (TS)	≥2 motor tics and ≥1 vocal tics	≥1 year	Symptoms persist without medicine or other drugs or due to having another medical condition.
Tic	Motor	Phonic	
Classification of tic	Description		
Simple	Abrupt and fleeting (typically < 1 s), one group of muscles (e.g., eye blinking, facial grimacing, head jerk, shoulder shrug)	Abrupt, maybe typecast, prolonged in duration, matched movements patterns.	
Complex	Sudden movements that appear purposive, stereotyped and are of a longer duration. Movements are coordinated.	Syllables, words, or phrases that may include odd patterns of speech. Alterations in rate, volume, or rhythm of words may be apparent.	
	- Echopraxia: repeating gestures of others. - Palipraxia: duplicating one’s own gestures. - Copropraxia: coarse and obscene gestures with hands or tongue. - Dystonic: constant, bending, or twisting movement or posture (e.g., blepharospasm, oculogyric movements, mouth opening, shoulder rotation). - Tonic: persistent, isometric muscle contraction - Self-injurious behavior: tics that include self-inflicted injury (e.g., lip biting or deliberate hitting of the face).	- Echolalia: repeating words or phrases of others - Palilalia: duplicating one’s own words or phrases. - Coprolalia: socially unacceptable syllables, words, or phrases voiced in a loud, volatile manner (e.g., swearing loudly and repeatedly)	

**Table 2:** Pain descriptors and muscular impact due to TS and tics (from Małek [37]).

Classification	Description
Exertional	Excessive muscular contraction
	Skeletal or joint pain
	Neuropathic pain (due to spinal cord, radicular or peripheral nerve compression)
Traumatic	Pain in a body part struck by a moving limb
	Pain in a moving body part striking something nearby
	Self-damage (self-inflicted)
	Pain from compulsive touching of hot or sharp objects
	And/or inflicted on others from tics or compulsions
Pain Caused by Tic Suppression	Due to persistent tics that can result in muscle strain, joint stress and/or harm or injury from the vigorous execution of movements.
Pain Relief	Pharmacological and non-pharmacological (medication, behavioral treatments, and psychological therapies).

## Data Availability

No new data were created or analyzed in this study.

## Abbreviations

The following abbreviations are used in this manuscript:

ADHD	Attention-Deficit Hyperactivity Disorder
CBIT	Comprehensive behavioral intervention for tics
CNS	Central Nervous System
CSTC	Cortico–striato–thalamo-cortical circuitry
DCI	Diagnostic Confidence Index
DTI	Diffusion tensor imaging
DSM-5	The American Psychiatric Diagnostic and Statistical Manual of Mental Disorders
OCD	Obsessive–compulsive disorder
SF-MPQ-Z	McGill Pain Questionnaire-2
TS	Tourette Syndrome
VAS	Visual Analog Scale
YGTSS	Yale Global Tic Severity Scale) and Visual Analog Scale
